# Periodic Solutions of Asymptotically Linear Autonomous Hamiltonian Systems with Resonance

**DOI:** 10.1007/s10884-017-9608-0

**Published:** 2017-08-10

**Authors:** Anna Gołȩbiewska

**Affiliations:** Faculty of Mathematics and Computer Science, Nicolaus Copernicus University, ul. Chopina 12/18, 87-100 Toruń, Poland

**Keywords:** Autonomous Hamiltonian systems, Bifurcation from infinity, Degree for G-invariant strongly indefinite functionals, Primary 34C25, Secondary 47H11

## Abstract

In this paper we define the index at infinity of an asymptotically linear autonomous Hamiltonian system. We use this index to prove the existence and bifurcation from infinity of periodic solutions of the system. We apply the degree for *G*-invariant strongly indefinite functionals defined by Gołȩbiewska and Rybicki in (Nonlinear Anal 74:1823–1834, 2011).

## Introduction

Consider the problem of existence of periodic solutions of the system

1.1 $$\begin{aligned} \dot{x}=JH'(x), \end{aligned}$$

where $H \in C^2(\mathbb {R}^{2N}, \mathbb {R})$ is such that $H'$ is asymptotically linear at infinity, i.e. $H'(x)=H''(\infty )x+o(|x|)$ for $|x|\rightarrow \infty ,$ where $H''(\infty )$ is a symmetric matrix.

One of the ideas of studying such a system is to consider an associated functional defined on an appropriate Hilbert space. Using this functional one can define an index of the stationary solution and of the infinity. Comparing these indices we can prove the existence of solutions. Such an idea has been used by many authors, see for example [1, 10, 15, 16, 21]. The methods used to define the indices include theories of Morse index and the Conley index.

In the paper [12] we have defined the indices, using the degree of $S^1$-invariant strongly indefinite functionals. Namely, we have considered the system (1.1) with assumptions

1. $$(H')^{-1}(0) =\{p_1, \ldots , p_q\},$$
2. $$\sigma (JH''(\infty )) \cap i\mathbb {Z}= \emptyset .$$

Assumption (2) implies the system is nonresonant at the infinity. On the other hand, the stationary solutions can be resonant. For *p* being such a solution and for the infinity we have defined indices (or almost all their coordinates) $I_H(p)$ and $I_H(\infty )$ being elements of the Euler ring $U(S^1)$. Comparing the sum of the indices $I_H(p)$ with the index $I_H(\infty )$ we proved the existence of solutions, see Theorems 3.1, 3.2 of [12].

The main aim of our paper is to define the index $I_H(\infty )$ in the resonant case. To this end, following the method of Su (see [20], also [4, 17]), we introduce the additional assumptions, see conditions (H4) and (H5) of Sect. 3, and obtain the so called strong angle conditions on the associated functional.

Note that the index $I_H(\infty )$ can be also used for studying other problems, for instance the bifurcation from infinity, i.e. the problem of the existence of unbounded closed connected sets of periodic solutions of the family of systems:

$$\begin{aligned} \dot{x}=JH'(x, \lambda ), \end{aligned}$$

where $H \in C^2(\mathbb {R}^{2N} \times \mathbb {R}, \mathbb {R})$ is such that $H'(x, \lambda )$ is asymptotically linear at infinity. It is known that if the difference of the indices computed on some levels $\lambda _-, \lambda _+$ is nontrivial, then there exists an unbounded continuum of solutions. The proof of this fact in the case of the operator being completely continous perturbation of the identity can be found in [8], the proof in the general case is analogous.

After this introduction this paper is organized in the following way: in Sect. 2 we fix notation and remind the definitions of degrees used in the next part of the paper. Moreover we compute the index at the infinity for the asymptotically linear operator. To this end we introduce the so called strong angle conditions.

In Sect. 3 we study periodic solutions of autonomous Hamiltonian systems. We formulate main results of this paper, namely Theorems 3.1 and 3.3. In the former one we prove the existence of solutions in the resonant case, while in the latter one we prove the existence of a connected set of solutions bifurcating from the infinity.

## Preliminaries

In this section we collect basic facts from the $S^1$-equivariant degree theory. We remind, for $G=S^1,$ the properties of the degree for *G*-equivariant gradient maps defined by Gȩba in [9]. We also recall for $G=S^1$ the generalisation of this degree defined in [13], namely the degree for $S^1$-invariant strongly indefinite functionals.

The degrees mentioned above are elements of the Euler ring $U(S^1)$. The definition and the properties of this ring in the general case of any compact Lie group *G* one can find for example in [6, 11]. It is known that $U(S^1)$ can be identified with the ring $\displaystyle {\mathbb {Z}\oplus \left( \bigoplus _{k=1}^{\infty } \mathbb {Z}\right) }$ with actions $+, \star : U(S^1) \times U(S^1) \rightarrow U(S^1)$ and $\cdot :\mathbb {Z}\times U(S^1)\rightarrow U(S^1)$ defined as follows:

2.1 $$\begin{aligned} \begin{aligned} \alpha +\beta&=(\alpha _0+\beta _0, \alpha _1+\beta _1, \ldots , \alpha _k+\beta _k, \ldots ),\\ \alpha \star \beta&=(\alpha _0\cdot \beta _0, \alpha _0\cdot \beta _1+\beta _0 \cdot \alpha _1, \ldots , \alpha _0\cdot \beta _k+\beta _0 \cdot \alpha _k, \ldots ), \\ \gamma \cdot \alpha&=(\gamma \cdot \alpha _0, \gamma \cdot \alpha _1, \ldots , \gamma \cdot \alpha _k, \ldots ), \end{aligned} \end{aligned}$$

for $\alpha =(\alpha _0, \alpha _1, \ldots , \alpha _k, \ldots ), \beta =(\beta _0, \beta _1, \ldots , \beta _k, \ldots ) \in U(S^1)$, $\gamma \in \mathbb {Z}.$ We set $\Theta = (0, 0, \ldots , 0, \ldots )$ and $\mathbb {I}=(1, 0, \ldots , 0, \ldots ).$ Note that we can index the coordinates of elements of $U(S^1)$ by the conjugacy classes of closed subgroups of $S^1$, writing $\alpha =(\alpha _{S^1}, \alpha _{\mathbb {Z}_1}, \ldots , \alpha _{\mathbb {Z}_k}, \ldots ).$

Let $\mathbb {V}$ be a real, orthogonal $S^1$-representation. Put $C^k_{S^1}(\mathbb {V}, \mathbb {R})=\{f \in C^k(\mathbb {V}, \mathbb {R}); f \text { is } S^1-\text {invariant}\}.$ Since $\mathbb {V}$ is an orthogonal representation, for $f \in C^k_{S^1}(\mathbb {V},\mathbb {R})$ the gradient $\nabla f:\mathbb {V}\rightarrow \mathbb {V}$ is an $S^1$-equivariant $C^{k-1}$-map.

For $\delta >0$ and $p \in \mathbb {V}$ we denote by $B_{\delta }(\mathbb {V}, p)$ an open ball centered in *p* with radius $\delta .$ We also write $B_{\delta }(\mathbb {V})$ instead of $B_{\delta }(\mathbb {V},0)$ and $B(\mathbb {V})$ instead of $B_1(\mathbb {V}).$

### Degree for $S^1$-Equivariant Gradient Maps

Let $\mathbb {V}$ be a finite-dimensional, orthogonal $S^1$-representation and $\Omega \subset \mathbb {V}$ an open, bounded and $S^1$-invariant subset. For $f \in C^1_{S^1}(\mathbb {V}, \mathbb {R})$ such that $(\nabla f)^{-1}(0) \cap \partial \Omega = \emptyset$ one can consider the degree $\nabla _{S^1}\text {-}{\mathrm {deg}}(\nabla f, \Omega ) \in U(S^1)$, as a special case of the degree for *G*-equivariant gradient maps defined by Gȩba in [9]. The coordinates of the degree can be written in the following way:

$$\begin{aligned} \nabla _{S^1}\text {-}{\mathrm {deg}}(\nabla f, \Omega )=(\nabla _{S^1}\text {-}{\mathrm {deg}}_{S^1}(\nabla f, \Omega ), \nabla _{S^1}\text {-}{\mathrm {deg}}_{\mathbb {Z}_1}(\nabla f, \Omega ), \ldots , \nabla _{S^1}\text {-}{\mathrm {deg}}_{\mathbb {Z}_k}(\nabla f, \Omega ),\ldots ). \end{aligned}$$

#### Remark 2.1

Note that the definition of the degree for $S^1$-equivariant, orthogonal maps has been given also in [19]. Since every gradient map is an orthogonal one, we can use this definition instead of the one mentioned above. However, formulas defining degree in those two approaches differ by sign. The general summary of the equivariant degree theory can be found in [2, 3].

The properties of the degree are formulated in the following theorem (see [9]):

#### Theorem 2.1

1. Let $\Omega$ and *f* satisfy the above assumptions. Then:
  - (Existence) If $\nabla _{S^1}\text {-}{\mathrm {deg}}(\nabla f, \Omega ) \ne \Theta \in U(S^1),$ then $(\nabla f)^{-1} (0) \cap \Omega \ne \emptyset .$
  - (Additivity) If $\Omega = \Omega _0 \cup \Omega _1$, where $\Omega _0, \Omega _1$ are open, disjoint, $S^1$-invariant sets, then $\nabla _{S^1}\text {-}{\mathrm {deg}}(\nabla f, \Omega ) = \nabla _{S^1}\text {-}{\mathrm {deg}}( \nabla f, \Omega _0)+\nabla _{S^1}\text {-}{\mathrm {deg}}(\nabla f, \Omega _1).$
  - (Excision) If $\Omega _0 \subset \Omega$ is an open, $S^1$-invariant subset and $(\nabla f)^{-1}(0) \cap \Omega \subset \Omega _0,$ then $\nabla _{S^1}\text {-}{\mathrm {deg}}(\nabla f, \Omega )= \nabla _{S^1}\text {-}{\mathrm {deg}}(\nabla f, \Omega _0).$
  - (Linearisation) If $f \in C^2_{S^1}(\mathbb {V}, \mathbb {R})$ is such that $\nabla f(0)=0$ and $\nabla ^2f(0)$ is an $S^1$-equivariant linear isomorphism, then there exists $\alpha _0>0$ such that for every $\alpha <\alpha _0$ we have $\nabla _{S^1}\text {-}{\mathrm {deg}}(\nabla f, B_{\alpha }(\mathbb {V}))=\nabla _{S^1}\text {-}{\mathrm {deg}}(\nabla ^2f(0), B(\mathbb {V})).$
2. (Homotopy invariance) Let $h\in C^1_G(\mathbb {V}\times [0,1], \mathbb {R})$ be such that $(\nabla _vh)^{-1}(0) \cap (\partial \Omega \times [0,1])=\emptyset .$ Then $\nabla _G\text {-}{\mathrm {deg}}(\nabla _vh(\cdot , 0), \Omega )=\nabla _G\text {-}{\mathrm {deg}}(\nabla _vh(\cdot , 1),\Omega ).$

To compute the degree of a product map we use for $G=S^1$ the following fact, proven in the general case in [14].

#### Fact 2.1

(Product formula) Let $\Omega _i \subset \mathbb {V}_i$ be an open, bounded, $S^1$-invariant subset of a finite-dimensional, orthogonal $S^1$-representation and $f_i \in C^1_{S^1}(\mathbb {V}_i, \mathbb {R})$ be such that $(\nabla f_i)^{-1}(0) \cap \partial \Omega _i =\emptyset$ for $i=1,2$. Then

$$\begin{aligned} \nabla _{S^1}\text {-}{\mathrm {deg}}((\nabla f_1, \nabla f_2), \Omega _1 \times \Omega _2)=\nabla _{S^1}\text {-}{\mathrm {deg}}(\nabla f_1, \Omega _1) \star \nabla _{S^1}\text {-}{\mathrm {deg}}(\nabla f_2, \Omega _2). \end{aligned}$$

Let us now remind the classification of the equivalence classes of finite-dimensional $S^1$-representations. Recall that two representations $\mathbb {V}, \mathbb {V}'$ are equivalent (briefly $\mathbb {V}\approx \mathbb {V}'$) if there exists an equivariant linear isomorphism $L:\mathbb {V}\rightarrow \mathbb {V}'.$ For $k \in \mathbb {N}$ define an $S^1$-action on $\mathbb {R}^2$ by $\gamma _t\cdot (x,y)=(x \cos (kt)-y \sin (kt), x \sin (kt)+y\cos (kt))$ where $\gamma _t=\cos t+i\sin t\in S^1, (x,y) \in \mathbb {R}^2.$ Denote by $\mathbb {R}[1,k]$ the two-dimensional $S^1$-representation with this action and put $\displaystyle {\mathbb {R}[j,k]=\bigoplus _{i=1}^j\mathbb {R}[1,k]}$ for $j \in \mathbb {N}$. Moreover denote by $\mathbb {R}[j,0]$ the trivial *j*-dimensional $S^1$-representation.

It is known that any finite-dimensional $S^1$-representation is equivalent to $\displaystyle {\bigoplus _{i=1}^r \mathbb {R}[j_i,k_i]}$ for some $r \in \mathbb {N}, j_i \in \mathbb {N}, k_i \in \mathbb {N}\cup \{0\}.$ Using this fact and the definition of the degree, we obtain the following computational formulas for the degree of a self-adjoint, $S^1$-equivariant linear isomorphism (see [9]). By $m^-(L)$ we denote the Morse index of *L*, i.e. the sum of multiplicities of negative eigenvalues of *L*.

#### Fact 2.2

Let $\mathbb {V}\approx \mathbb {R}[j_0,0] \oplus \mathbb {R}[j_1,k_1] \oplus \ldots \oplus \mathbb {R}[j_r,k_r],$ where $j_0 \in \mathbb {N}\cup \{0\}, j_1, \ldots , j_r$, $k_1, \ldots , k_r \in \mathbb {N}$ and let $L:\mathbb {V}\rightarrow \mathbb {V}$ be a self-adjoint, $S^1$-equivariant linear isomorphism. Then

1. $$L={\mathrm { diag \;}}(L_0, L_1, \ldots , L_r),$$ where $$L_i:\mathbb {R}[j_i, k_i]\rightarrow \mathbb {R}[j_i, k_i]$$ for $$i=0, \ldots , r,$$
2. $$\nabla _{S^1}\text {-}{\mathrm {deg}}_H(L, B(\mathbb {V}))={\left\{ \begin{array}{ll} (-1)^{m^-(L_0)} &{} \text { for } H=S^1,\\ (-1)^{m^-(L_0)+1}\cdot \dfrac{m^-(L_i)}{2} &{}\text { for }H=\mathbb {Z}_{k_i},\\ 0 &{}\text { for } H \notin \{S^1, \mathbb {Z}_{k_1}, \ldots , \mathbb {Z}_{k_r}\}. \end{array}\right. }$$
3. In particular, if $L=-Id,$ then
  $$\begin{aligned} \nabla _{S^1}\text {-}{\mathrm {deg}}_H(-Id, B(\mathbb {V}))={\left\{ \begin{array}{ll} (-1)^{j_0} &{} \text { for } H=S^1,\\ (-1)^{j_0+1}\cdot j_i &{}\text { for }H=\mathbb {Z}_{k_i},\\ 0 &{}\text { for } H \notin \{S^1, \mathbb {Z}_{k_1}, \ldots , \mathbb {Z}_{k_r}\}. \end{array}\right. } \end{aligned}$$

#### Remark 2.2

Note that if $L :\mathbb {V}\rightarrow \mathbb {V}$ is an $S^1$-equivariant linear isomorphism, then the degree $\nabla _{S^1}\text {-}{\mathrm {deg}}(L, B(\mathbb {V}))$ is invertible. Moreover

$$\begin{aligned} (\pm 1, \alpha _1, \ldots , \alpha _k, \ldots )^{-1} = (\pm 1, -\alpha _1, \ldots , -\alpha _k, \ldots ). \end{aligned}$$

### Degree for $S^1$-Invariant Strongly Indefinite Functional

We briefly recall, in the special case $G=S^1$, the definition of the degree for *G*-invariant strongly indefinite functionals given in [13].

Let $(\mathbb {H}, \langle \cdot , \cdot \rangle _{\mathbb {H}})$ be an infinite-dimensional, separable Hilbert space, which is an orthogonal $S^1$-representation. Denote by $\Gamma =\{\pi _n: \mathbb {H}\rightarrow \mathbb {H}: n \in \mathbb {N}\cup \{0\}\}$ an $S^1$-equivariant approximation scheme on $\mathbb {H}$, i.e. a sequence of $S^1$-equivariant orthogonal projections satisfying

1. $\mathbb {H}^n=\pi _n(\mathbb {H})$ is a finite-dimensional subrepresentation, for all $n \in \mathbb {N}$,
2. there exists a subrepresentation $\mathbb {H}_n$ of $\mathbb {H}^{n+1}$ such that $\mathbb {H}^{n+1}=\mathbb {H}^n \oplus \mathbb {H}_n$ and $\mathbb {H}^n \perp \mathbb {H}_n$ for all $n \in \mathbb {N}$,
3. $$\displaystyle {\lim _{n\rightarrow \infty } \pi _n(u)=u}$$ for all $$u \in \mathbb {H}.$$

Assume that:

1. $\Omega \subset \mathbb {H}$ is an open, bounded, $S^1$-invariant set,
2. $L:\mathbb {H}\rightarrow \mathbb {H}$ is a linear, bounded, self-adjoint, $S^1$-equivariant Fredholm operator of index 0, such that $\ker L=\mathbb {H}^0$ and $\pi _n \circ L=L \circ \pi _n$ for all $n \in \mathbb {N}\cup \{0\},$
3. $\mathcal {K}\in C^1_{S^1}(cl(\Omega ), \mathbb {R})$ is such that $\nabla \mathcal {K}: cl(\Omega ) \rightarrow \mathbb {H}$ is an $S^1$-equivariant completely continuous operator,
4. $\Phi \in C^1_{S^1}(cl(\Omega ), \mathbb {R})$ is of the form $\Phi (u)=\frac{1}{2} \langle Lu,u\rangle _{\mathbb {H}} - \mathcal {K}(u),$
5. $$(\nabla \Phi )^{-1} (0) \cap \partial \Omega = \emptyset .$$

Put $\mathcal {Z}=(\nabla \Phi )^{-1}(0)$, $r_0={\mathrm { dist \;}}(\mathcal {Z}, \partial \Omega )$ and $\Omega _{\varepsilon }=\mathcal {Z}+B_{\varepsilon }(\mathbb {H}),$ for $\varepsilon \le \frac{r_0}{3}.$

#### Definition 2.1

Let $\Phi \in C^1_{S^1}(cl(\Omega ), \mathbb {R})$ satisfy assumptions (a1)–(a5). The degree for $S^1$-invariant strongly indefinite functionals, denoted by $\nabla _{S^1}\text {-}{\mathrm {deg}}(\nabla \Phi , \Omega ) \in U(S^1)$ is defined by formula

$$\begin{aligned} \nabla _{S^1}\text {-}{\mathrm {deg}}(\nabla \Phi , \Omega ) = \nabla _{S^1}\text {-}{\mathrm {deg}}(L, B(\mathbb {H}^n\ominus \mathbb {H}^0))^{-1} \star \nabla _{S^1}\text {-}{\mathrm {deg}}(L-\pi _n\circ \nabla \mathcal {K}, \Omega _{\varepsilon } \cap \mathbb {H}^n), \end{aligned}$$

where *n* is sufficiently large and $\varepsilon$ sufficiently small.

It was shown in [13] that such the degree is well-defined and it has the same properties as the degree for $S^1$-equivariant gradient mappings, i.e. properties of existence, additivity, excision, linearisation and homotopy invariance. In the fact below we formulate the slightly different version of the last of those properties, the so called generalized homotopy invariance property. The proof of this fact carries over from the Leray-Schauder degree case, see [5]. We put an assumption:

(a6) $\Phi \in C^1_{S^1}(\mathbb {H}\times \mathbb {R}, \mathbb {R})$ is such that $\Phi (u,\lambda )=\frac{1}{2}\langle Lu,u\rangle _{\mathbb {H}}-\mathcal {K}(u,\lambda ),$ where *L* satisfies (a2) and $\nabla _u \mathcal {K}$ is an $S^1$-equivariant completely continuous operator.

#### Fact 2.3

Let $\Phi :\mathbb {H}\times \mathbb {R}\rightarrow \mathbb {R}$ satisfy condition (a6) and $\tilde{\Omega } \subset \mathbb {H}\times [\lambda _-, \lambda _+]$ be an open, bounded and $S^1$-invariant subset. Moreover assume that there exist open, bounded, $S^1$-invariant sets $\tilde{\Omega }_+, \tilde{\Omega }_- \subset \mathbb {H}$ such that

1. $$\tilde{\Omega }\cap (\mathbb {H}\times \{\lambda _{\pm }\})=\tilde{\Omega }_{\pm } \times \{\lambda _{\pm }\},$$
2. $$(\nabla _u\Phi )^{-1}(0) \cap \partial \tilde{\Omega } \subset (\tilde{\Omega }_- \times \{\lambda _-\}) \cup (\tilde{\Omega }_+ \times \{\lambda _+\}).$$

Then $$\nabla _{S^1}\text {-}{\mathrm {deg}}(\nabla _u\Phi (\cdot ,\lambda _-), \tilde{\Omega }_{-})=\nabla _{S^1}\text {-}{\mathrm {deg}}(\nabla _u\Phi (\cdot ,\lambda _+), \tilde{\Omega }_{+}).$$

Using this property we can study the bifurcation from infinity of solutions of the equation $\nabla _u\Phi (u,\lambda )=0.$ Assume that there exist $\lambda _-, \lambda _+ \in \mathbb {R}$ satisfying $\lambda _-<\lambda _+$ and $\gamma >0$ such that

2.2 $$\begin{aligned} (\nabla _u\Phi (\cdot , \lambda _{\pm }))^{-1}(0) \subset B_{\gamma }(\mathbb {H}). \end{aligned}$$

Define the bifurcation index at $\infty$ for $[\lambda _-, \lambda _+]$ by

$$\begin{aligned} {\mathrm {BIF}}(\infty , [\lambda _-, \lambda _+])=\nabla _{S^1}\text {-}{\mathrm {deg}}(\nabla _u\Phi (\cdot , \lambda _+), B_{\gamma }(\mathbb {H}))-\nabla _{S^1}\text {-}{\mathrm {deg}}(\nabla _u\Phi (\cdot , \lambda _-), B_{\gamma }(\mathbb {H})). \end{aligned}$$

#### Theorem 2.2

Let $\Phi$ satisfy condition (a6) and let $\lambda _{\pm } \in \mathbb {R}, \gamma >0$ be such that assumption (2.2) is fulfilled. If ${\mathrm {BIF}}(\infty , [\lambda _-, \lambda _+])\ne \Theta \in U(S^1)$, then there exists an unbounded closed connected component *C* of $(\nabla _u\Phi )^{-1}(0) \cap (\mathbb {H}\times [\lambda _-, \lambda _+])$ such that $C \cap (B_{\gamma }(\mathbb {H}) \times \{\lambda _-, \lambda _+\}) \ne \emptyset .$

The proof of this theorem in the case of the *SO*(2)-equivariant operators of the form compact perturbation of identity can be found in [8]. The authors use the properties of the *SO*(2)-degree, especially the generalized homotopy invariance property and the fact that the set $\nabla _u \Phi ^{-1}(0)$ is compact in $\mathbb {H}\times \mathbb {R}$. Using the counterparts of these facts in the case of the degree for *G*-invariant strongly indefinite functionals, we obtain our result.

### Index at the Infinity

In this section, under some additional assumptions, we compute the degree of an asymptotically linear operator, which is a gradient of a strongly indefinite, $S^1$-invariant functional at the sufficiently large disc centered at the origin. Let $(\mathbb {H}, \langle \cdot , \cdot \rangle _{\mathbb {H}})$ be an infinite dimensional Hilbert space, which is an orthogonal $S^1$-representation. Let $\Phi \in C^2_{S^1}(\mathbb {H},\mathbb {R})$ be such that

2.3 $$\begin{aligned} \Phi (u) = \frac{1}{2} \langle (L+L_{\infty })u,u\rangle _{\mathbb {H}} +\eta (u), \end{aligned}$$

where *L* satisfies condition (a2) of Sect. 2.2 and moreover

1. $L_{\infty }:\mathbb {H}\rightarrow \mathbb {H}$ is a linear, $S^1$-equivariant, self-adjoint, completely continuous operator, satisfying $L_{\infty } \circ \pi _n = \pi _n \circ L_{\infty },$
2. $\nabla \eta :\mathbb {H}\rightarrow \mathbb {H}$ is an $S^1$-equivariant, completely continuous operator,
3. $$\nabla \eta (u)=o(\Vert u\Vert _{\mathbb {H}})$$ as $$\Vert u\Vert _{\mathbb {H}} \rightarrow \infty .$$

Denote by $\nabla ^2\Phi (\infty )=L+L_{\infty }$ the linearization of $\nabla \Phi$ at the infinity. In the case when $\nabla ^2\Phi (\infty )$ is an isomorphism, analogously to the linearization property at the origin, one can prove the property of the linearization at the infinity.

#### Fact 2.4

Let $\Phi \in C^2_{S^1}(\mathbb {H}, \mathbb {R})$ be a functional given by (2.3) and let conditions (a2), (b1)–(b3) be satisfied. Assume that $\nabla ^2\Phi (\infty ):\mathbb {H}\rightarrow \mathbb {H}$ is an $S^1$-equivariant, self-adjoint isomorphism. Then there exists $\alpha _{\infty }>0$ such that for all $\alpha >\alpha _{\infty }$

$$\begin{aligned} \nabla _{S^1}\text {-}{\mathrm {deg}}(\nabla \Phi , B_{\alpha }(\mathbb {H}))=\nabla _{S^1}\text {-}{\mathrm {deg}}(\nabla ^2\Phi (\infty ),B(\mathbb {H})). \end{aligned}$$

In the rest of this section we will assume $\nabla ^2 \Phi (\infty )$ is not an isomorphism, i.e.

(b4) $$0 \in \sigma (\nabla ^2 \Phi (\infty ))$$.

Denote by $\mathbb {V}_{\infty }$ and $\mathbb {W}_{\infty }$ the kernel and the image of $\nabla ^2 \Phi (\infty )$, respectively and put $A_{\infty }=(\nabla ^2 \Phi (\infty ))_{|\mathbb {W}_{\infty }}:\mathbb {W}_{\infty } \rightarrow \mathbb {W}_{\infty }.$ Moreover, denote by $\mathbb {W}_{\infty }^+$ and $\mathbb {W}_{\infty }^-$ subspaces of $\mathbb {W}_{\infty }$ such that $(A_{\infty })_{|\mathbb {W}_{\infty }^+}$ is positive definite and $(A_{\infty })_{|\mathbb {W}_{\infty }^-}$ is negative definite.

To compute the degree of $\Phi$ at the sufficiently large disc centered at the origin we put additional assumptions, so called strong angle conditions.

(SAC$$_{\infty }^+$$)
There exists $M>0, \beta >0$ and $\alpha \in (0,\frac{\pi }{2})$ such that $\langle \nabla \Phi (u),\frac{v}{\Vert v\Vert _{\mathbb {H}}} \rangle _{\mathbb {H}} \ge \beta >0$ for any $u=(v,w) \in \mathbb {H}=\mathbb {V}_{\infty }\oplus \mathbb {W}_{\infty }$ with $\Vert u\Vert _{\mathbb {H}} \ge M$ and $\Vert w\Vert _{\mathbb {H}} \le \Vert u\Vert _{\mathbb {H}} \sin \alpha .$
(SAC$$_{\infty }^-$$)
There exists $M>0, \beta >0$ and $\alpha \in (0,\frac{\pi }{2})$ such that $\langle \nabla \Phi (u),\frac{v}{\Vert v\Vert _{\mathbb {H}}} \rangle _{\mathbb {H}} \le -\beta <0$ for any $u=(v,w) \in \mathbb {H}=\mathbb {V}_{\infty }\oplus \mathbb {W}_{\infty }$ with $\Vert u\Vert _{\mathbb {H}} \ge M$ and $\Vert w\Vert _{\mathbb {H}} \le \Vert u\Vert _{\mathbb {H}} \sin \alpha .$

The above conditions has been introduced by Li and Su in [17], see also [20]. Using the method introduced by Bartsch and Li in [4] for a similar type of assumptions (namely the angle conditions $(AC^{\pm }_{\infty }$) they have computed the critical groups of the functional at the infinity. Combining some arguments from [4] and the degree theory for $S^1$-invariant strongly indefinite functionals, we can compute the degree $\nabla _{S^1}\text {-}{\mathrm {deg}}(\nabla \Phi , B_{\gamma }(\mathbb {H})).$

#### Theorem 2.3

Let $\Phi \in C^2_{S^1}(\mathbb {H},\mathbb {R})$ be a functional given by (2.3) and let conditions (a2), (b1)–(b4) be satisfied. Then

- If $\Phi$ satisfies the condition (SAC$_{\infty }^+$) then for $\gamma$ and *n* sufficiently large
  $$\begin{aligned} \nabla _{S^1}\text {-}{\mathrm {deg}}(\nabla \Phi , B_{\gamma }(\mathbb {H}))= \nabla _{S^1}\text {-}{\mathrm {deg}}(L,B(\mathbb {H}^n \ominus \mathbb {H}^0))^{-1}\star \nabla _{S^1}\text {-}{\mathrm {deg}}(A_{\infty },B_{\gamma }(\mathbb {H}^n \ominus \mathbb {V}_{\infty })). \end{aligned}$$
- If $\Phi$ satisfies the condition (SAC$_{\infty }^-$) then for $\gamma$ and *n* sufficiently large
  $$\begin{aligned} \begin{aligned} \nabla _{S^1}\text {-}{\mathrm {deg}}(\nabla \Phi , B_{\gamma }(\mathbb {H}))&=\nabla _{S^1}\text {-}{\mathrm {deg}}(-Id,B_{\gamma }(\mathbb {V}_{\infty }))\star \nabla _{S^1}\text {-}{\mathrm {deg}}(L,B(\mathbb {H}^n \ominus \mathbb {H}^0))^{-1}\star \\&\quad ~~~\nabla _{S^1}\text {-}{\mathrm {deg}}(A_{\infty },B_{\gamma }(\mathbb {H}^n \ominus \mathbb {V}_{\infty })). \end{aligned} \end{aligned}$$

#### Proof

(a) We first prove that if $\Phi$ satisfies conditions (a2), (b1)–(b4) and ($\hbox {SAC}_{\infty }^+$), the conditions (a1)–(a5) of Sect. 2.2 are satisfied for $\Omega =B_{\gamma }(\mathbb {H})$, where $\gamma$ is sufficiently large. Therefore the degree $\nabla _{S^1}\text {-}{\mathrm {deg}}(\nabla \Phi , B_{\gamma }(\mathbb {H}))$ is well-defined. Indeed, the conditions (a1)–(a4) follows immediately from (a2), (b1)–(b4) and the definition of $\Omega .$ To show (a5) we will prove that for *M* sufficiently large if $\Vert u\Vert _{\mathbb {H}}>M$ then $\nabla \Phi (u) \ne 0$.

Choose $M>0$ and $\alpha \in (0, \frac{\pi }{2})$ as in ($\hbox {SAC}_{\infty }^+$)  and note that for $u=(v,w)\in \mathbb {V}_{\infty } \oplus \mathbb {W}_{\infty }= \mathbb {H}$ satisfying $\Vert u\Vert _{\mathbb {H}}>M$ and $\Vert w\Vert _{\mathbb {H}}\le \Vert u\Vert _{\mathbb {H}} \sin \alpha$, from ($\hbox {SAC}_{\infty }^+$) we obtain $\langle \nabla \Phi (u),v\rangle _{\mathbb {H}}>0.$ In the case $\Vert u\Vert _{\mathbb {H}} >M$ and $\Vert w\Vert _{\mathbb {H}} > \Vert u\Vert _{\mathbb {H}}\sin \alpha ,$ choose $\varepsilon >0$ such that $\frac{\varepsilon }{\sin \alpha } <\Vert A_{\infty }^{-1}\Vert ^{-1}$ and note that from (b3) without loss of generality we can assume that for $\Vert u\Vert _{\mathbb {H}}>M$ we have $\Vert \nabla \eta (u)\Vert _{\mathbb {H}}<\varepsilon \Vert u\Vert _{\mathbb {H}}.$ We obtain $\Vert \nabla \Phi (u)\Vert _{\mathbb {H}} \ge \Vert A_{\infty }^{-1}\Vert ^{-1} \Vert w\Vert _{\mathbb {H}} - \Vert \nabla \eta (u)\Vert _{\mathbb {H}} \ge (\Vert A_{\infty }^{-1}\Vert ^{-1} -\varepsilon \frac{1}{\sin \alpha })\Vert w\Vert _{\mathbb {H}}>0$.

To compute the degree define the homotopy $H^{\infty } \in C^2(\mathbb {H}\times [0,1], \mathbb {R})$ by the formula $H^{\infty }((v,w),t)=\Phi (v,w)+\frac{1}{2} t\Vert v\Vert _{\mathbb {H}}^2.$ We will show that this homotopy satisfies assumptions of Fact 2.3. Using arguments as in the proof of Proposition 2.5 in [4], one can show that for $\gamma$ sufficiently large and $u \in \mathbb {H}$ satisfying $\Vert u\Vert _{\mathbb {H}}> \gamma$:

1. if $\Vert w\Vert _{\mathbb {H}}\le \Vert u\Vert _{\mathbb {H}}\sin \alpha$ then from ($\hbox {SAC}_{\infty }^+$) follows $\Vert \nabla _uH^{\infty }(u,t)\Vert _{\mathbb {H}}^2>0$
2. if $\Vert w\Vert _{\mathbb {H}} > \Vert u\Vert _{\mathbb {H}} \sin \alpha ,$ where $w=(w^+,w^-)\in \mathbb {W}_{\infty }^+\oplus \mathbb {W}_{\infty }^-$ satisfies $\Vert w^+\Vert _{\mathbb {H}}\ge \Vert w^-\Vert _{\mathbb {H}}$ then using (b1) and (b3) we obtain $\langle \nabla _u H^{\infty }(u,t),w^+\rangle _{\mathbb {H}}>0,$
3. if $\Vert w\Vert _{\mathbb {H}} > \Vert u\Vert _{\mathbb {H}} \sin \alpha ,$ where $w=(w^+,w^-)\in \mathbb {W}_{\infty }^+\oplus \mathbb {W}_{\infty }^-$ satisfies $\Vert w^+\Vert _{\mathbb {H}}\le \Vert w^-\Vert _{\mathbb {H}}$ then using (b1) and (b3) we obtain $\langle \nabla _uH^{\infty }(u,t),w^-\rangle _{\mathbb {H}} <0.$

Hence we obtain that $\nabla _u H^{\infty }(u,t) \ne 0$ for every $u \in \mathbb {H}$ satisfying $\Vert u\Vert _{\mathbb {H}}>\gamma$ and $t \in [0,1].$ Moreover, $\nabla _u H^{\infty }(u,t)=L(u)-\nabla \psi (u,t),$ where $\psi (u,t)=\frac{1}{2}\langle L_{\infty }u,u\rangle _{\mathbb {H}}+\frac{1}{2} t\Vert v\Vert _{\mathbb {H}}^2-\eta (u).$ From assumptions (b1), (b2) and the fact that $t\cdot Id_{\mathbb {V}_{\infty }}$ is a finite dimensional mapping for every $t\in [0,1]$, we obtain that $\nabla \psi$ is $S^1$-equivariant and completely continuous. Therefore assumptions of the homotopy invariance property (see Fact 2.3) are satisfied. Hence:

2.4 $$\begin{aligned} \begin{aligned} \nabla _{S^1}\text {-}{\mathrm {deg}}(\nabla \Phi , B_{\gamma }(\mathbb {H}))&=\nabla _{S^1}\text {-}{\mathrm {deg}}(\nabla _u H^{\infty }(\cdot ,0),B_{\gamma }(\mathbb {H}))=\nabla _{S^1}\text {-}{\mathrm {deg}}(\nabla _u H^{\infty }(\cdot ,1),B_{\gamma }(\mathbb {H}))\\&=\nabla _{S^1}\text {-}{\mathrm {deg}}(\nabla \Phi +Id_{\mathbb {V}_{\infty }},B_{\gamma }(\mathbb {H})). \end{aligned} \end{aligned}$$

Note that functional $\Psi :\mathbb {H}\rightarrow \mathbb {R}$ defined by $\Psi (v,w)=\Phi (v,w)+\frac{1}{2}\Vert v\Vert _{\mathbb {H}}^2$ satisfies assumptions of the property of linearization at the infinity (Fact 2.4). Using this fact and Definition 2.1 we have

2.5 $$\begin{aligned} \begin{aligned}&\nabla _{S^1}\text {-}{\mathrm {deg}}(\nabla \Phi +Id_{\mathbb {V}_{\infty }}, B_{\gamma }(\mathbb {H}))=\nabla _{S^1}\text {-}{\mathrm {deg}}(L+L_{\infty }+Id_{\mathbb {V}_{\infty }},B_{\gamma }(\mathbb {H}))\\&\quad =\nabla _{S^1}\text {-}{\mathrm {deg}}(L,B(\mathbb {H}^n\ominus \mathbb {H}^0))^{-1}\star \nabla _{S^1}\text {-}{\mathrm {deg}}(L+\pi _n\circ (L_{\infty }+Id_{\mathbb {V}_{\infty }}), (B_{\gamma }(\mathbb {H}))_{\varepsilon }\cap \mathbb {H}^n), \end{aligned} \end{aligned}$$

where, according to the definition of the degree, $(B_{\gamma }(\mathbb {H}))_{\varepsilon }$ is an $\varepsilon$-neighbourhood of the set $(L+L_{\infty }+Id_{\mathbb {V}_{\infty }})^{-1}(0)\cap B_{\gamma }(\mathbb {H}).$ Since $L+L_{\infty }+Id_{\mathbb {V}_{\infty }}$ is an isomorphism, $(B_{\gamma }(\mathbb {H}))_{\varepsilon }\cap \mathbb {H}^n=B_{\varepsilon }(\mathbb {H}^n).$

For *n* sufficiently large, from (a2) and (b1), $\mathbb {V}_{\infty } \subset \mathbb {H}^n.$ Therefore, using the product formula,

2.6 $$\begin{aligned} \begin{aligned}&\nabla _{S^1}\text {-}{\mathrm {deg}}(L+\pi _n\circ (L_{\infty }+Id_{\mathbb {V}_{\infty }}), (B_{\gamma }(\mathbb {H}))_{\varepsilon }\cap \mathbb {H}^n)\\&\quad =\nabla _{S^1}\text {-}{\mathrm {deg}}((Id_{\mathbb {V}_{\infty }},(A_{\infty })_{|\mathbb {H}^n\ominus \mathbb {V}_{\infty }}),B_{\varepsilon }(\mathbb {H}^n))\\&\quad =\nabla _{S^1}\text {-}{\mathrm {deg}}(Id_{\mathbb {V}_{\infty }}, B_{\varepsilon }(\mathbb {V}_{\infty }))\star \nabla _{S^1}\text {-}{\mathrm {deg}}(A_{\infty },B_{\varepsilon }(\mathbb {H}^n \ominus \mathbb {V}_{\infty })). \end{aligned} \end{aligned}$$

From (2.4)–(2.6), we obtain the assertion.

To prove (b) it is enough to consider the homotopy $H^{\infty }\in C^2(\mathbb {H}\times [0,1],\mathbb {R})$ defined by the formula $H^{\infty }((v,w),t)=\Phi (v,w)-\frac{1}{2}t\Vert v\Vert ^2_{\mathbb {H}}.$ The rest of the proof is analogous to the proof of (a). $\square$

#### Remark 2.3

Note that if $L(\mathbb {V}_{\infty }) \subset \mathbb {V}_{\infty }$, from the excision property of the degree and the product formula we have

$$\begin{aligned} \begin{aligned}&\nabla _{S^1}\text {-}{\mathrm {deg}}(L, B(\mathbb {H}^n \ominus \mathbb {H}^0))^{-1}\\&\quad =\nabla _{S^1}\text {-}{\mathrm {deg}}(L, B(\mathbb {H}^n\ominus (\mathbb {V}_{\infty }\oplus \mathbb {H}^0)))^{-1} \star \nabla _{S^1}\text {-}{\mathrm {deg}}(L, B(\mathbb {V}_{\infty } \ominus \mathbb {H}^0))^{-1}. \end{aligned} \end{aligned}$$

Therefore, using again the definition of the degree, we have

$$\begin{aligned} \nabla _{S^1}\text {-}{\mathrm {deg}}(\nabla \Phi , B_{\gamma }(\mathbb {H}))=\nabla _{S^1}\text {-}{\mathrm {deg}}(L, B_{\gamma }(\mathbb {V}_{\infty } \ominus \mathbb {H}^0))^{-1} \star \nabla _{S^1}\text {-}{\mathrm {deg}}(A_{\infty }, B_{\gamma }(\mathbb {W}_{\infty })). \end{aligned}$$

## Periodic Solutions of Autonomous Hamiltonian Systems

Throughout this section we study the existence of periodic solutions of autonomous Hamiltonian systems of the form:

3.1 $$\begin{aligned} \dot{x}=JH'(x), \end{aligned}$$

where $$J:=\left( \begin{array}{cc} 0 &{}\quad -Id\\ Id &{}\quad 0 \end{array}\right) $$ and $H \in C^2(\mathbb {R}^{2N}, \mathbb {R})$ satisfies

1. $H'(x)=H''(\infty )x+g'(x)=H''(\infty )x+o(|x|)$ for $|x|\rightarrow \infty ,$ where $H''(\infty )$ is a symmetric matrix,
2. $$(H')^{-1}(0)=\{p_1, \ldots , p_q\}.$$

We also study the problem of existence of closed connected sets of periodic solutions bifurcating from infinity for the family of autonomous Hamiltonian systems:

3.2 $$\begin{aligned} \dot{x}=JH'(x,\lambda ), \end{aligned}$$

where $$H \in C^2(\mathbb {R}^{2N} \times \mathbb {R},\mathbb {R})$$ satisfies

(H3) $H'(x,\lambda )=H''(\infty ,\lambda )x+g'(x,\lambda )=H''(\infty ,\lambda )x+o(|x|)$ for $|x| \rightarrow \infty$ uniformly on bounded $\lambda$-intervals, where $H''(\infty ,\lambda )$ is a real, symmetric matrix for all $\lambda \in \mathbb {R}$.

### Remark 3.1

Note that if $H \in C^2(\mathbb {R}^{2N} \times \mathbb {R}, \mathbb {R})$ satisfies (H3), then for a fixed $\lambda$ the potential $H_{\lambda } \in C^2(\mathbb {R}^{2N}, \mathbb {R})$ defined by $H_{\lambda }(x)=H(x,\lambda )$ satisfies condition (H1) with $H''(\infty )=H''(\infty , \lambda ).$

We start with recalling the definitions of the appropriate Hilbert space and the functional corresponding to this system. Put $\mathbb {E}:=\mathbb {H}^{1/2}(S^1, \mathbb {R}^{2N}),$ the Sobolev space of functions $u(t)=a_0+\sum _{k=1}^{\infty }(a_k\cos kt+b_k \sin kt),$ satisfying $\sum _{k=1}^{\infty }k(|a_k|^2+|b_k|^2)<\infty ,$ where $a_0,a_k,b_k \in \mathbb {R}^{2N}$.

It is known that $\mathbb {E}$ is a separable Hilbert space with an inner product defined by the formula

3.3 $$\begin{aligned} \langle u,u'\rangle _{\mathbb {E}} :=2\pi a_0 \cdot a_0'+\pi \sum _{k=1}^{\infty } k(a_k \cdot a_k'+b_k\cdot b_k'). \end{aligned}$$

Moreover, if we consider an $S^1$-action given by $\gamma \cdot u(t)=u(t+\varphi )$ for $\gamma =\cos \varphi +i \sin \varphi \in S^1, u \in \mathbb {E}$, it is easy to show that $\mathbb {E}$ is an orthogonal representation of the group $S^1$.

Define a sequence of projections $\Gamma =\{\pi _n:\mathbb {E}\rightarrow \mathbb {E}; \; n \in \mathbb {N}\cup \{0\}\}$ by $\pi _n(a_0+\sum _{k=1}^{\infty }(a_k\cos kt+b_k \sin kt))=a_0+\sum _{k=1}^n(a_k \cos kt+b_k \sin kt)$ and put $\mathbb {E}^n=\pi _n(\mathbb {E}).$ Then $\Gamma$ is an $S^1$-equivariant approximation scheme.

Under the assumption (H1) (or (H3) respectively) one can prove (see [18]) that $u(t) \in C^2(\mathbb {R}, \mathbb {R}^{2N})$ is a $2\pi$-periodic solution of (3.1) ((3.2) respectively) if and only if *u* is a critical point with respect to *u* of the functional

3.4 $$\begin{aligned} \Phi _H(u)=\frac{1}{2}\int _0^{2\pi } J \dot{u}(t)\cdot u(t)+ \int _0^{2\pi } H(u(t))dt, \end{aligned}$$

or respectively

3.5 $$\begin{aligned} \tilde{\Phi }_H(u,\lambda )=\frac{1}{2}\int _0^{2\pi } J \dot{u}(t)\cdot u(t)+ \int _0^{2\pi } H(u(t),\lambda )dt. \end{aligned}$$

Moreover $\Phi _H \in C^2_{S^1}(\mathbb {E},\mathbb {R}), \tilde{\Phi }_H \in C^2_{S^1}(\mathbb {E}\times \mathbb {R}, \mathbb {R}).$ Let us summarize the properties of these functionals.

Define $\mathcal {L}: \mathbb {E}\times \mathbb {E}\rightarrow \mathbb {R}$ by $\mathcal {L}(u,v)=\int _0^{2\pi } J\dot{u}(t) \cdot v(t)dt$. From the Riesz Theorem we obtain the existence of a unique, bounded, $S^1$-equivariant, self-adjoint Fredholm operator of index 0, $L:\mathbb {E}\rightarrow \mathbb {E}$ such that $\langle Lu,v\rangle _{\mathbb {E}}=\mathcal {L}(u,v).$

Using the definition of *L* and the inner product formula (3.3) we obtain an explicit formula for this operator. Namely for $u=a_0+\sum _{k=1}^{\infty }(a_k \cos kt+b_k \sin kt)$ we have:

3.6 $$\begin{aligned} Lu=\sum _{k=1}^{\infty }(Jb_k \cos kt-Ja_k \sin kt). \end{aligned}$$

From the above we obtain

1. $$\ker L=\pi _0(\mathbb {E}),$$
2. $$\pi _n\circ L=L \circ \pi _n$$ for all $$n \in \mathbb {N}\cup \{0\}.$$

Define $S^1$-representations $\mathbb {E}_k^+, \mathbb {E}_k^-, \mathbb {E}_k$ by $\mathbb {E}_k^{\pm }:=\{a \cos kt \mp Ja \sin kt; \; a \in \mathbb {R}^{2N}\}$ and $\mathbb {E}_k=\mathbb {E}_k^+\oplus \mathbb {E}_k^-.$ Moreover let $\mathbb {E}_0=\mathbb {R}^{2N} \approx \mathbb {R}[2N,0].$ It is easy to show that $\mathbb {E}_k^{\pm } \approx \mathbb {R}[N,k], \mathbb {E}_k\approx \mathbb {R}[2N,k]$ and $L_{|\mathbb {E}_k^+}=Id, L_{|\mathbb {E}_k^-}=-Id.$ Moreover, $\mathbb {E}^n = \bigoplus _{k=0}^n \mathbb {E}_k.$

Note that using (H1) and (H3) we can rewrite $\Phi _H$ and $\tilde{\Phi }_H$ as

$$\begin{aligned} \begin{aligned} \Phi _H(u)&=\frac{1}{2}\langle Lu,u\rangle _{\mathbb {E}}+\frac{1}{2} \int _0^{2\pi } H''(\infty )u(t) \cdot u(t) dt+\int _0^{2\pi } g(u(t))dt,\\ \tilde{\Phi }_H(u, \lambda )&=\frac{1}{2}\langle Lu,u\rangle _{\mathbb {E}} +\frac{1}{2} \int _0^{2\pi } H''(\infty , \lambda ) u(t) \cdot u(t) dt+\int _0^{2\pi }g(u(t), \lambda )dt. \end{aligned} \end{aligned}$$

From the Riesz theorem it follows that there exists a unique, bounded linear operator $L_{\infty }:\mathbb {E}\rightarrow \mathbb {E}$ defined by

3.7 $$\begin{aligned} \langle L_{\infty }u,v\rangle _{\mathbb {E}}=\int _0^{2\pi } H''(\infty )u(t) \cdot v(t) dt. \end{aligned}$$

Additionally we put $\eta (u)=\int _0^{2\pi } g(u(t))dt.$ It is easy to check that $L_{\infty }$ and $\nabla \eta$ are $S^1$-equivariant and, since $H''(\infty )$ is symmetric, $L_{\infty }$ is self-adjoint. Moreover, it is known (see [18]) that $L_{\infty }$ and $\nabla \eta$ are completely continuous and that condition $g'(x)=o(|x|)$ for $|x| \rightarrow \infty$ implies $\nabla \eta (u)=o(\Vert u\Vert _{\mathbb {E}})$ as $\Vert u\Vert _{\mathbb {E}} \rightarrow \infty$ (see [16]).

Let *A* be a real, symmetric, $(2N \times 2N)$-matrix. Consider the functional associated to a linear system

3.8 $$\begin{aligned} \dot{x}=JAx. \end{aligned}$$

According to (3.4) we obtain the functional $\Phi _A:\mathbb {E}\rightarrow \mathbb {R}$ given by

$$\begin{aligned} \Phi _A(u)=\frac{1}{2}\langle Lu,u\rangle _{\mathbb {E}}+\frac{1}{2}\langle Bu,u\rangle _{\mathbb {E}}, \text { where } \langle Bu,v\rangle _{\mathbb {E}}=\int _0^{2\pi } Au(t)\cdot v(t) dt. \end{aligned}$$

From the above definition and (3.3) we can compute the explicit formula for *B*. Namely, for $u=a_0+\sum _{k=1}^{\infty }(a_k \cos kt+b_k \sin kt)\in \mathbb {E}$ we have

3.9 $$\begin{aligned} Bu=Aa_0+\sum _{k=1}^{\infty }\left( \frac{1}{k}(Aa_k) \cos kt+ \frac{1}{k} (Ab_k) \sin kt\right) . \end{aligned}$$

### Remark 3.2

Note that from (3.9) it follows that $\pi _n \circ B = B \circ \pi _n$ for $n \in \mathbb {N}\cup \{0\}.$

### Corollary 3.1

From the above considerations, taking $A=H''(\infty )$, we obtain that the functional $\Phi _H$ is of the form (2.3) with conditions (a2),(b1)–(b3) satisfied. The same statement is valid if we consider $\tilde{\Phi }_{H}(\cdot , \lambda )$ with $\lambda$ fixed.

For $k \in \mathbb {N}$ put $$T_k(A):=\left[ \begin{array}{cc} \frac{1}{k}A &{}\quad J\\ -J &{}\quad \frac{1}{k}A \end{array} \right] .$$ Note that from (3.6) and (3.9) it follows that the restriction of $\nabla \Phi _A$ to $\mathbb {E}_k$ can be represented by the matrix $T_k(A)$ for $k\ge 1$ and by *A* for $k=0.$

From the above considerations we obtain the following properties of the operator $\nabla \Phi _A$.

### Lemma 3.1

1. $\nabla \Phi _A$ is an isomorphism if and only if $\sigma (JA)\cap i\mathbb {Z}=\emptyset .$
2. $$\displaystyle {\ker \nabla \Phi _A=\ker A \oplus \bigoplus _{k=1}^{\infty } \ker (T_k(A))}$$ $$\displaystyle {=\{u \equiv a; \; a \in \ker A\} \oplus \bigoplus _{k=1}^{\infty }\{u_k=a_k \cos kt+ b_k \sin kt ; \; a_k+ib_k \in V_{JA}(-ik)\}}$$.
3. $\displaystyle {\ker \nabla \Phi _A \approx \bigoplus _{k=0}^{\infty } \mathbb {R}[m_k,k]}$, where $m_k$ is a geometric multiplicity of an eigenvalue *ik* of the matrix *JA*.
4. $$\displaystyle {\bigoplus _{\lambda _i<0} \mathbb {V}_{\nabla \Phi _A}(\lambda _i) \approx \mathbb {R}[m^-(A),0]\oplus \bigoplus _{k=1}^{\infty }\mathbb {R}[m^-(T_k(A)),k]}.$$

In the case of an isomorphism, we have the following formula for the degree of $\nabla \Phi _A$, see [12].

### Lemma 3.2

If $$\sigma (JA)\cap i \mathbb {Z}=\emptyset ,$$ then

$$\begin{aligned} \begin{aligned}&\nabla _{S^1}\text {-}{\mathrm {deg}}(\nabla \Phi _A,B(\mathbb {E}))\\&\quad =\left( (-1)^{m^-(A)},(-1)^{m^-(A)}\cdot \left( N-\frac{m^-(T_1(A))}{2}\right) , \ldots , (-1)^{m^-(A)}\cdot \left( N-\frac{m^-(T_k(A))}{2}\right) , \ldots \right) . \end{aligned} \end{aligned}$$

For a nonlinear Hamiltonian system with potential *H* satisfying (H1) we define an index $I_H(\infty ) \in U(S^1)$ depending on the matrix $H''(\infty ).$ We start with a nonresonant case.

### Definition 3.1

Let *H* be such that $\sigma (JH''(\infty ))\cap i\mathbb {Z}=\emptyset$. Define $I_H(\infty ) \in U(S^1)$ by

$$\begin{aligned} I_H(\infty )_K= {\left\{ \begin{array}{ll} (-1)^{m^-(H''(\infty ))}&{}\text { for } K=S^1,\\ (-1)^{m^-(H''(\infty ))}\left( N-\frac{m^-(T_k(H''(\infty )))}{2}\right) &{}\text { for }K=\mathbb {Z}_k. \end{array}\right. } \end{aligned}$$

### Remark 3.3

Note that for $k \in \mathbb {N}$ sufficiently large, $m^-(T_k(H''(\infty )))=2N$, hence $I_H(\infty )$ is a well-defined element of $U(S^1).$

### Lemma 3.3

Let *H* satisfy condition (H1) and $\sigma (JH''(\infty ))\cap i\mathbb {Z}= \emptyset$. Then, for $\gamma$ sufficiently large, $I_H(\infty )=\nabla _{S^1}\text {-}{\mathrm {deg}}(\nabla \Phi _H, B_{\gamma }(\mathbb {E})).$

### Proof

The proof follows from the linearization property at the infinity and Lemma 3.2. $\square$

### Remark 3.4

Note that for $p \in (H')^{-1}(0)$ satisfying $\sigma (JH''(p)) \cap i\mathbb {Z}=\emptyset$ one can define the index $I_H(p)\in U(S^1)$ analogously as $I_H(\infty )$, see Definition 3.1 of [12]. Moreover, in this case $\nabla _{S^1}\text {-}{\mathrm {deg}}(\nabla \Phi _H, B_{\alpha }(\mathbb {E},p))=I_H(p)$ for $\alpha >0$ sufficiently small, see Lemma 3.3 of [12]. If $\sigma (JH''(p)) \cap i\mathbb {Z}= \{\pm ik_1, \ldots , \pm ik_m\}$, we can define $I_H(p),$ or almost all its coordinates, by

$$\begin{aligned} \begin{aligned}&I_H(p)_K\\&\quad = {\left\{ \begin{array}{ll} \displaystyle {\lim _{\alpha \rightarrow 0}} \mathrm {deg}_B (JH', B_{\alpha }(\mathbb {R}^{2N},p),0), &{}\text {for } K=S^1,\\ \displaystyle {\lim _{\alpha \rightarrow 0}} \mathrm {deg}_B (JH', B_{\alpha }(\mathbb {R}^{2N},p),0)\cdot \left( N-\frac{m^-(T_k(H''(p)))}{2}\right) , &{}\text {for } K=\mathbb {Z}_k, k \notin \mathbb {K}(m) \end{array}\right. } \end{aligned} \end{aligned}$$

where $\mathbb {K}(m)=\bigcup _{\{i_1, \ldots , i_s\}\subset \{1, \ldots , m\}}\{\gcd (k_{i_1}, \ldots , k_{i_s})\}$, see Definition 3.2 of [12]. Moreover, for *K* such that $I_H(p)_K$ is defined, $I_H(p)_K=\nabla _{S^1}\text {-}{\mathrm {deg}}_K(\nabla \Phi _H,B_{\alpha }(\mathbb {E},p))$ for $\alpha$ sufficiently small, see Theorem 3.2 of [12].

In the following we define an index $I_H(\infty )$ in the case when $\sigma (JH''(\infty ))\cap i\mathbb {Z}\ne \emptyset$. We assume that one of the following additional conditions on potential *H* is satisfied. Remind that $H'(x)=H''(\infty )x+g'(x).$

(H4) There exist $R,c_1,c_2>0,$ and $0<s<1$ such that for all $x \in \mathbb {R}^{2N}$ $(g'(x),x) \le 0$ and moreover for all $x \in \mathbb {R}^{2N}$ with $|x| \ge R$ we have
  $$\begin{aligned} |(g'(x),x)| \ge c_1|x|^{s+1}, |g'(x)|\le c_2|x|^s. \end{aligned}$$
(H5) There exist $R,c_1,c_2>0$ and $0<s<1$ such that for all $x \in \mathbb {R}^{2N}$ with $|x|\ge R$ we have
  $$\begin{aligned} (g'(x),x)\ge c_1|x|^{s+1}, |g'(x)|\le c_2|x|^s. \end{aligned}$$

### Remark 3.5

The above conditions have been introduced by Su, see [20]. Moreover, if *H* satisfies (H4) then the functional $\Phi _H$ satisfies ($\hbox {SAC}_{\infty }^+$), and if *H* satisfies (H5) then $\Phi _H$ satisfies ($\hbox {SAC}_{\infty }^-$), see Lemma 3.1 of [20].

### Definition 3.2

Let *H* satisfy (H1), $\sigma (JH''(\infty ))\cap i\mathbb {Z}\ne \emptyset$ and condition (H4) be satisfied. Define $I_H(\infty ) \in U(S^1)$ by

$$\begin{aligned} I_H(\infty )_K= {\left\{ \begin{array}{ll} (-1)^{m^-(H''(\infty ))}&{}\text { for } K=S^1,\\ (-1)^{m^-(H''(\infty ))}\left( N-\frac{m^-(T_k(H''(\infty )))}{2}\right) &{}\text { for }K=\mathbb {Z}_k. \end{array}\right. } \end{aligned}$$

Note that $I_H(\infty )$ is a well-defined element of $U(S^1).$

### Lemma 3.4

Let *H* satisfy condition (H1) and $\sigma (JH''(\infty ))\cap i\mathbb {Z}\ne \emptyset$. Moreover assume that (H4) is satisfied. Then, for $\gamma$ sufficiently large, $I_H(\infty )=\nabla _{S^1}\text {-}{\mathrm {deg}}(\nabla \Phi _H, B_{\gamma }(\mathbb {E})).$

### Proof

From Corollary 3.1, Lemma 3.1 and Remark 3.5 we obtain that $\Phi _H$ satisfies conditions (a2), (b1)-(b4) and (SAC$_{\infty }^+$). Therefore, using Theorem 2.3, we have, for $\gamma$ and *n* sufficiently large,

3.10 $$\begin{aligned} \nabla _{S^1}\text {-}{\mathrm {deg}}(\nabla \Phi _H,B_{\gamma }(\mathbb {E}))= \nabla _{S^1}\text {-}{\mathrm {deg}}(L, B(\mathbb {E}^n \ominus \mathbb {E}^0))^{-1} \star \nabla _{S^1}\text {-}{\mathrm {deg}}(\nabla ^2\Phi _H(\infty ), B_{\gamma }(\mathbb {E}^n \ominus \mathbb {V}_{\infty })), \end{aligned}$$

where $\mathbb {V}_{\infty }$ is the kernel of the operator $\nabla ^2\Phi _H(\infty ).$

From the product formula and the definition of representations $\mathbb {E}_k^+, \mathbb {E}_k^-$, we have

$$\begin{aligned} \nabla _{S^1}\text {-}{\mathrm {deg}}(L, B(\mathbb {E}^n \ominus \mathbb {E}^0))^{-1} = \nabla _{S^1}\text {-}{\mathrm {deg}}(-Id, B(\mathbb {E}^-_1))^{-1} \star \ldots \star \nabla _{S^1}\text {-}{\mathrm {deg}}(-Id, B(\mathbb {E}^-_n))^{-1}. \end{aligned}$$

Theorefore, since representations $\mathbb {E}^{-}_k$ are equivalent to $\mathbb {R}[N,k],$ from Fact 2.2 and Remark 2.2 it follows that

$$\begin{aligned} \nabla _{S^1}\text {-}{\mathrm {deg}}_K(L, B(\mathbb {E}^n \ominus \mathbb {E}^0))^{-1}={\left\{ \begin{array}{ll} 1 &{}\text { for } K=S^1,\\ N &{}\text { for } K=\mathbb {Z}_i, i\le n,\\ 0 &{}\text { for } K=\mathbb {Z}_i, i>n. \end{array}\right. } \end{aligned}$$

To compute the latter factor of (3.10), note that the operator $\nabla ^2\Phi _H(\infty )$ is associated to the linear equation $\dot{x}=JH''(\infty )x.$ Therefore, from Lemma 3.1 we conclude that $\mathbb {V}_{\infty } = \ker (H''(\infty ))\oplus \bigoplus _{k=1}^{\infty } \ker (T_k(H''(\infty )))$. Moreover $m^-((\nabla ^2\Phi _H(\infty ))_{|\mathbb {E}_k \cap \mathbb {W}_{\infty }})=m^-(T_k(H''(\infty ))),$ where $\mathbb {W}_{\infty }=\mathbb {H}\ominus \mathbb {V}_{\infty }.$ Hence, Fact 2.2 yields:

$$\begin{aligned} \begin{aligned}&\nabla _{S^1}\text {-}{\mathrm {deg}}_K(\nabla ^2\Phi _H(\infty ),B_{\gamma }(\mathbb {E}^n \ominus \mathbb {V}_{\infty }))\\&\quad ={\left\{ \begin{array}{ll} (-1)^{m^-(H''(\infty ))} &{}\text { for } K=S^1,\\ (-1)^{m^-(H''(\infty ))+1}\cdot \frac{m^-(T_k(H''(\infty )))}{2} &{}\text { for }K=\mathbb {Z}_k, k\le n,\\ 0 &{}\text { for } K=\mathbb {Z}_k, k>n. \end{array}\right. } \end{aligned} \end{aligned}$$

Using formulae (2.1) we obtain the assertion. $\square$

Denote by $\tilde{m}^-(A)$ the sum of multiplicities of nonpositive eigenvalues of the symmetric matrix *A*.

### Definition 3.3

Let *H* satisfy (H1), $\sigma (JH''(\infty ))\cap i\mathbb {Z}\ne \emptyset$ and condition (H5) be satisfied. Define $I_H(\infty ) \in U(S^1)$ by

$$\begin{aligned} I_H(\infty )_K= {\left\{ \begin{array}{ll} (-1)^{\tilde{m}^-(H''(\infty ))}&{}\text { for } K=S^1,\\ (-1)^{\tilde{m}^-(H''(\infty ))}\left( N-\frac{\tilde{m}^-(T_k(H''(\infty )))}{2}\right) &{}\text { for }K=\mathbb {Z}_k. \end{array}\right. } \end{aligned}$$

### Lemma 3.5

Let *H* satisfy (H1) and $\sigma (JH''(\infty ))\cap i\mathbb {Z}\ne \emptyset$. Moreover assume that condition (H5) is satisfied. Then, for $\gamma$ sufficiently large, $I_H(\infty )=\nabla _{S^1}\text {-}{\mathrm {deg}}(\nabla \Phi _H, B_{\gamma }(\mathbb {E})).$

### Proof

From Corollary 3.1, Lemma 3.1 and Remark 3.5 we obtain that $\Phi _H$ satisfies conditions (a2), (b1)-(b4) and (SAC$_{\infty }^-$). Therefore, using Theorem 2.3, we have, for $\gamma$ and *n* sufficiently large,

$$\begin{aligned} \begin{aligned} \nabla _{S^1}\text {-}{\mathrm {deg}}(\nabla&\Phi _H,B_{\gamma }(\mathbb {E}))=\nabla _{S^1}\text {-}{\mathrm {deg}}(-Id,B_{\gamma }(\mathbb {V}_{\infty }))\\&\quad \star \nabla _{S^1}\text {-}{\mathrm {deg}}(L,B(\mathbb {E}^n \ominus \mathbb {E}^0))^{-1}\star \nabla _{S^1}\text {-}{\mathrm {deg}}(\nabla \Phi _H(\infty ),B_{\gamma }(\mathbb {E}^n \ominus \mathbb {V}_{\infty })), \end{aligned} \end{aligned}$$

where $\mathbb {V}_{\infty }$ is the kernel of the operator $\nabla ^2\Phi _H(\infty )$ associated to the linear equation $\dot{x}=JH''(\infty )x.$ Observe that from Lemma 3.1(3) $\mathbb {V}_{\infty }\approx \bigoplus _{k=0}^{\infty } \mathbb {R}[m_k,k]$, where $m_k$ is a geometric multiplicity of an eigenvalue *ik* of the matrix $JH''(\infty ).$ Therefore, from Fact 2.2,

$$\begin{aligned} \nabla _{S^1}\text {-}{\mathrm {deg}}(-Id, B_{\gamma }(\mathbb {V}_{\infty }))=((-1)^{m_0},(-1)^{m_0+1}\cdot m_1, \ldots , (-1)^{m_0+1}\cdot m_k, \ldots ). \end{aligned}$$

Reasoning as in the proof of the previous lemma, we obtain

$$\begin{aligned} \begin{aligned}&\nabla _{S^1}\text {-}{\mathrm {deg}}(\nabla \Phi _H, B_{\gamma }(\mathbb {E}))\\&\quad =\left( (-1)^{m_0+m^-(H''(\infty ))}, \ldots , (-1)^{m_0+m^-(H''(\infty ))}\left( N-\frac{m^-(T_k(H''(\infty )))}{2}-m_k\right) , \ldots \right) . \end{aligned} \end{aligned}$$

To end the proof we use the fact that the pair of the eigenvalues $ik, -ik$ of the matrix $JH''(\infty )$ corresponds to an eigenvalue 0 of matrix $T_k(H''(\infty )).$ Therefore $2m_k$ is the multiplicity of the eigenvalue 0 and $m^-(T_k(H''(\infty )))+2m_k=\tilde{m}^-(T_k(H''(\infty )))$. $\square$

### Existence of Solutions

In the following we use the definition of the index $I_H(\infty )$ to formulate conditions sufficient to the existence of solutions of (3.1).

#### Theorem 3.1

Let $H \in C^2(\mathbb {R}^{2N}, \mathbb {R})$ satisfy assumptions (H1), (H2) and $\sigma (JH''(p)) \cap i\mathbb {Z}=\emptyset$ for all $p \in (H')^{-1}(0).$ Moreover, let $\sigma (JH''(\infty )) \cap i\mathbb {Z}\ne \emptyset$ and one of the conditions (H4), (H5) be satisfied. If $I_H(\infty )\ne \sum _{i=1}^q I_H(p_i)$ then there exists at least one non-stationary $2\pi$-periodic solution of (3.1).

#### Proof

Consider the functional $\Phi _H$ given by (3.4). From Lemma 3.1 follows that if *p* is a critical point of *H* satisfying $\sigma (JH''(p)) \cap i\mathbb {Z}=\emptyset$, then the solution $u \equiv p$ is an isolated critical point of $\Phi _H$. Moreover, since *H* satisfy (H4) or (H5), and therefore $\Phi _H$ satisfies (SAC$_{\infty }^+$) or (SAC$_{\infty }^-$), the set $(\nabla \Phi _H)^{-1}(0)$ is bounded, see the proof of Theorem 2.3. Therefore, we can choose $\alpha _{p_i}, \alpha _{\infty }>0$ for $i=1, \ldots , q,$ such that $B_{\alpha _{p_i}}(\mathbb {E}, p_i)$ are disjoint neighborhoods of $p_i \in \mathbb {E}$ satisfying $cl(B_{\alpha _{p_i}}(\mathbb {E}, p_i)) \subset B_{\alpha _{\infty }}(\mathbb {E})$ for $i=1, \ldots , q.$ Suppose, contrary to our claim, that $p_1, \ldots , p_q$ are the only $2\pi$-periodic solutions of (3.1). From the additivity and the excision properties of the degree, we have

$$\begin{aligned} \nabla _{S^1}\text {-}{\mathrm {deg}}(\nabla \Phi _H, B_{\alpha _{\infty }}(\mathbb {E}))=\sum _{i=1}^q\nabla _{S^1}\text {-}{\mathrm {deg}}(\nabla \Phi _H,B_{\alpha _{p_i}}(\mathbb {E}, p_i)). \end{aligned}$$

Taking into consideration Remark 3.4, Lemmas 3.4 and 3.5, we obtain

$$\begin{aligned} I_H(\infty )=\sum _{i=1}^q I_H(p_i), \end{aligned}$$

a contradiction. $$\square $$

In the following corollaries, using the definition of $I_H(\infty )$, we formulate the assertion of the Theorem 3.1 in terms of Morse indices of matrices $H''(\infty ), T_k(H''(\infty )).$

#### Corollary 3.2

Let $H \in C^2(\mathbb {R}^{2N}, \mathbb {R})$ satisfy assumptions (H1), (H2) and $\sigma (JH''(p)) \cap i\mathbb {Z}=\emptyset$ for all $p \in (H')^{-1}(0).$ Moreover, let $\sigma (JH''(\infty )) \cap i\mathbb {Z}\ne \emptyset$ and the condition (H4) be satisfied. Assume that one of the following conditions is fulfilled:

1. $$(-1)^{m^-(H''(\infty ))} \ne \sum _{i=1}^q (-1)^{m^-(H''(p_i))},$$
2. $$ (-1)^{m^-(H''(\infty ))} \left( N-\frac{m^-(T_k(H''(\infty )))}{2}\right) \ne \sum _{i=1}^q (-1)^{m^-(H''(p_i))}\left( N-\frac{m^-(T_k(H''(p_i)))}{2}\right) $$ for some $$k \in \mathbb {N}$$.

Then there exists at least one non-stationary $2\pi$-periodic solution of (3.1).

#### Corollary 3.3

The above corollary remains valid if we replace (H4) by (H5) and conditions (1), (2) by:

1. $$(-1)^{\tilde{m}^-(H''(\infty ))} \ne \sum _{i=1}^q (-1)^{m^-(H''(p_i))},$$
2. $$ (-1)^{\tilde{m}^-(H''(\infty ))} \left( N-\frac{\tilde{m}^-(T_k(H''(\infty )))}{2}\right) \ne \sum _{i=1}^q (-1)^{m^-(H''(p_i))}\left( N-\frac{m^-(T_k(H''(p_i)))}{2}\right) $$ for some $$k \in \mathbb {N}$$.

Note that we can omit the assumption $\sigma (JH''(p))\cap i\mathbb {Z}=\emptyset$ for $p \in (H')^{-1}(0)$ by defining the index $I_H(p)$ (or almost all its coordinates) as in Remark 3.4. In such a situation we obtain the following theorem:

#### Theorem 3.2

Let $H \in C^2(\mathbb {R}^{2N}, \mathbb {R})$ satisfy assumptions (H1), (H2), $\sigma (JH''(\infty )) \cap i\mathbb {Z}\ne \emptyset$ and one of the conditions (H4), (H5) is fulfilled. Let $k \in \mathbb {Z}$ be such that for every $p \in \{p_1, \ldots , p_q\}$ the number $I_H(p)_{\mathbb {Z}_k}$ is defined. If $I_H(\infty )_{\mathbb {Z}_k} \ne \sum _{k=1}^q I_H(p_i)_{\mathbb {Z}_k}$ then there exists at least one non-stationary $2\pi$-periodic solution of (3.1).

#### Proof

Observe, that we can assume $p_1,\ldots , p_q$ are isolated critical points of the functional $\Phi _H$. If not, we obtain an infinite sequence of $2\pi$-periodic solutions of (3.1) and hence the assertion of the theorem.

Therefore the degrees $\nabla _{S^1}\text {-}{\mathrm {deg}}(\nabla \Phi _H, B_{\alpha _{\infty }}(\mathbb {E})), \nabla _{S^1}\text {-}{\mathrm {deg}}(\nabla \Phi _H, B_{\alpha _{p_i}}(\mathbb {E}, p_i))$ are well-defined. Moreover, for $k \in \mathbb {Z}$ such that $I_H(p)_{\mathbb {Z}_k}$ is defined, $I_H(p)_{\mathbb {Z}_k}=\nabla _{S^1}\text {-}{\mathrm {deg}}_{\mathbb {Z}_k}(\nabla \Phi _H,B_{\alpha }(\mathbb {E},p)).$ The rest of the proof is analogous to the proof of Theorem 3.1. $\square$

### Bifurcation from Infinity

Let us now study the problem of bifurcation from infinity of solutions of the family (3.2). Assume that $\lambda _-<\lambda _+$ are such that for $\lambda \in \{\lambda _-, \lambda _+\}$ one of the following conditions is satisfied:

(A$$\lambda $$)
$$\sigma (JH''(\infty , \lambda ))\cap i\mathbb {Z}=\emptyset $$,
(B$$\lambda $$)
$\sigma (JH''(\infty , \lambda ))\cap i\mathbb {Z}\ne \emptyset$ and for $H=H(\cdot , \lambda )$ the condition (H4) is satisfied,
(C$$\lambda $$)
$\sigma (JH''(\infty , \lambda ))\cap i\mathbb {Z}\ne \emptyset$ and for $H=H(\cdot , \lambda )$ the condition (H5) is satisfied.

#### Theorem 3.3

Consider the family (3.2), where $H \in C^2(\mathbb {R}^{2N} \times \mathbb {R}, \mathbb {R})$ is such that condition (H3) is satisfied. Let $\lambda _-<\lambda _+$. Assume that one of the following conditions is satisfied:

- *(BIF1)* For $\lambda \in \{\lambda _-, \lambda _+\}$ one of the conditions (A$\lambda$), (B$\lambda$) is satisfied and moreover $(-1)^{m^-(H''(\infty , \lambda _-))} \ne (-1)^{m^-(H''(\infty , \lambda _+))}$ or there exists $k \in \mathbb {Z}$ such that $(-1)^{m^-(H''(\infty , \lambda _-))}\left( N-\frac{m^-(T_k(H''(\infty , \lambda _-)))}{2}\right) \ne (-1)^{m^-(H''(\infty , \lambda _+))}\left( N-\frac{m^-(T_k(H''(\infty , \lambda _+)))}{2}\right) .$
- *(BIF2)* For $\lambda =\lambda _-$ one of the conditions (A$\lambda$), (B$\lambda$) is satisfied and for $\lambda =\lambda _+$ the condition (C$\lambda$) is satisfied. Moreover $(-1)^{m^-(H''(\infty , \lambda _-))} \ne (-1)^{\tilde{m}^-(H''(\infty , \lambda _+))}$ or there exists $k \in \mathbb {Z}$ such that $(-1)^{m^-(H''(\infty , \lambda _-))}\left( N-\frac{m^-(T_k(H''(\infty , \lambda _-)))}{2}\right) \ne (-1)^{\tilde{m}^-(H''(\infty , \lambda _+))}\left( N-\frac{\tilde{m}^-(T_k(H''(\infty , \lambda _+)))}{2}\right) .$ *(BIF3)* For $\lambda =\lambda _-$ the condition (C$\lambda$) is satisfied and for $\lambda =\lambda _+$ one of the conditions (A$\lambda$), (B$\lambda$) is satisfied. Moreover $(-1)^{\tilde{m}^-(H''(\infty , \lambda _-))} \ne (-1)^{{m}^-(H''(\infty , \lambda _+))}$ or there exists $k \in \mathbb {Z}$ such that $(-1)^{\tilde{m}^-(H''(\infty , \lambda _-))}\left( N-\frac{\tilde{m}^-(T_k(H''(\infty , \lambda _-)))}{2}\right) \ne (-1)^{{m}^-(H''(\infty , \lambda _+))}\left( N-\frac{{m}^-(T_k(H''(\infty , \lambda _+)))}{2}\right) .$
- *(BIF4)* For $\lambda \in \{\lambda _-, \lambda _+\}$ the condition (C$\lambda$) is satisfied and moreover $(-1)^{\tilde{m}^-(H''(\infty , \lambda _-))} \ne (-1)^{\tilde{m}^-(H''(\infty , \lambda _+))}$ or there exists $k \in \mathbb {Z}$ such that $(-1)^{\tilde{m}^-(H''(\infty , \lambda _-))}\left( N-\frac{\tilde{m}^-(T_k(H''(\infty , \lambda _-)))}{2}\right) \ne (-1)^{\tilde{m}^-(H''(\infty , \lambda _+))}\left( N-\frac{\tilde{m}^-(T_k(H''(\infty , \lambda _+)))}{2}\right) .$

Then there exist $\gamma >0$ and an unbounded, closed, connected set $C \subset \mathbb {E}\times [\lambda _-, \lambda _+]$ of solutions of the family (3.2) such that $C \cap (B_{\gamma }(\mathbb {E}) \times \{\lambda _-, \lambda _+\})\ne \emptyset .$

#### Proof

Consider the functional $\tilde{\Phi }_H$ given by (3.5). Since *H* satisfies condition (H3), following the idea given in [16] we can prove that $\tilde{\Phi }_H$ satisfies (a6) of Sect. 2.2. Moreover, if the condition (A$\lambda$) is fulfilled then $\nabla _u\tilde{\Phi }_H(\cdot , \lambda )$ is an isomorphism satisfying (b1)–(b3) of Sect. 2.3. In the case of condition (B$\lambda$) (respectively (C$\lambda$)), from Corollary 3.1, Lemma 3.1 and Remark 3.5 follows that $\tilde{\Phi }_H(\cdot , \lambda )$ satisfies (b1)–(b4) and ($\hbox {SAC}_{\infty }^-$) (respectively ($\hbox {SAC}_{\infty }^+$)). Hence if one of the conditions (A$\lambda$), (B$\lambda$), (C$\lambda$) holds, then

$$\begin{aligned} (\nabla _u\tilde{\Phi }_H(\cdot , \lambda ))^{-1}(0) \subset B_{\gamma }(\mathbb {H}). \end{aligned}$$

For the proof in the case (B$\lambda$), (C$\lambda$), see the proof of Theorem 2.3. Therefore using the arguments as in the proofs of Lemmas 3.3–3.5 we can show that if one of the conditions (BIF1)–(BIF4) is satisfied then $BIF(\infty , [\lambda _-, \lambda _+]) \ne \Theta .$ Using Theorem 2.2 we obtain the assertion. $\square$
